# Mushroom Body Extrinsic Neurons in Walking Bumblebees Correlate With Behavioral States but Not With Spatial Parameters During Exploratory Behavior

**DOI:** 10.3389/fnbeh.2020.590999

**Published:** 2020-10-20

**Authors:** Nanxiang Jin, Benjamin H. Paffhausen, Aron Duer, Randolf Menzel

**Affiliations:** Institut für Biologie – Neurobiologie, Freie Universität Berlin, Berlin, Germany

**Keywords:** bumblebee, mushroom body extrinsic neurons, behavioral states, exploratory behavior, artificial arena environment

## Abstract

Central place foraging insects like honeybees and bumblebees learn to navigate efficiently between nest and feeding site. Essential components of this behavior can be moved to the laboratory. A major component of navigational learning is the active exploration of the test arena. These conditions have been used here to search for neural correlates of exploratory walking in the central arena (ground), and thigmotactic walking in the periphery (slope). We chose mushroom body extrinsic neurons (MBENs) because of their learning-related plasticity and their multi-modal sensitivities that may code relevant parameters in a brain state-dependent way. Our aim was to test whether MBENs code space-related components or are more involved in state-dependent processes characterizing exploration and thigmotaxis. MBENs did not respond selectively to body directions or locations. Their spiking activity differently correlated with walking speed depending on the animals’ locations: on the ground, reflecting exploration, or on the slope, reflecting thigmotaxis. This effect depended on walking speed in different ways for different animals. We then asked whether these effects depended on spatial parameters or on the two states, exploration and thigmotaxis. Significant epochs of stable changes in spiking did not correlate with restricted locations in the arena, body direction, or walking transitions between ground and slope. We thus conclude that the walking speed dependencies are caused by the two states, exploration and thigmotaxis, rather than by spatial parameters.

## Introduction

Animals exposed to a novel environment perform typical searching movements during which they explore the environment. Exploration is an active form of learning about the features of the environment that calibrates the reference systems for moving throughout space and time. Social animals are particularly dependent on exploratory learning since they need to return reliably and safely to their home sites. Social bees like honeybees and bumble bees exhibit multiple forms of exploratory learning both for local navigation and for way-finding over greater distances (Srinivasan et al., 2004; Collett et al., 2006; Menzel, 2017). Landmarks need to be distinguished and used for navigation with respect to their spatial properties and identities.

Neural systems for navigation are well studied in mammals (Rowland et al., 2016), but little is known in insects. The central complex has been related to the sun compass (Homberg, 2004; Collett, 2019; Currier and Nagel, 2020), and the mushroom body (MB) to multiple forms of learning about object identities (Menzel, 2014). In most of these studies the animals were not able to actively explore the environment, rather they were restricted to experimental conditions that allowed researchers to combine neural recordings with tests of innate control mechanisms and Pavlovian forms of associative learning (Menzel et al., 2007). Active exploration, however, is a major component in navigation and needs to be included in the search for neural correlates as it is the case in studies with rats (Moser et al., 2008), bats (Ulanovsky and Moss, 2007), and cockroaches (Mizunami et al., 1998b). Spatial restriction of the usually small test area often leads to switches between exploration of the open space and escape behavior as indicated by thigmotactic runs along the confining walls. It is, therefore, essential to separate neural correlates of spatial coding from state-dependent behavioral states that cause the animal to switch between exploration and thigmotaxis.

Several attempts have been made in the past to include operant behavior in the search for high order integration processes in the honeybee. Neural correlates were found for social interactions and active exploration-like behavior (Duer et al., 2015; Paffhausen et al., 2020). Active visual learning could be transferred at least partially to a virtual reality condition in which the bee performed stationary walking on an air-supported ball and controlled the visual environment (Buatois et al., 2018). Mushroom body extrinsic neurons (MBENs) were recorded under these conditions (Zwaka et al., 2019) and neural correlates of operant forms of visual learning could be demonstrated. Most MBENs form dense banding patterns in restricted zones in the vertical lobe and project into the calyx, pedunculus, β-lobe, and the protocerebral lobe (Rybak and Menzel, 1993). MBENs are known to respond to multiple sensory conditions in a integrative or combinatorial way and they are sensitive to the context in which stimuli appear (Filla and Menzel, 2015; Zwaka et al., 2018). We thus expected neural correlates for exploration in MBENs.

Bumble bees are more cooperative than honeybees in experimental setups that aim to move essential components of navigation into the laboratory (Jin et al., 2014). Most interestingly they guided their walks with respect to only the panorama. As expected, they also showed thigmotactic escape behavior during which they did not guide their walking trajectories to local or panorama cues. Similarly, in the present study bumblebees performed exploratory runs in a small round arena experiencing for the first time a novel environment consisting of an unstructured ground plate. Bumblebees walked actively on the arena ground covering most of the space in multiple trajectories and ran along the slope when trying to escape. This arrangement allowed us to address the question whether the neural activity of the recorded MBENs correlated with spatial experience during active exploration or with the behavioral states of exploration and thigmotactic escape.

## Materials and Methods

### Animals

**FIGURE 1:**
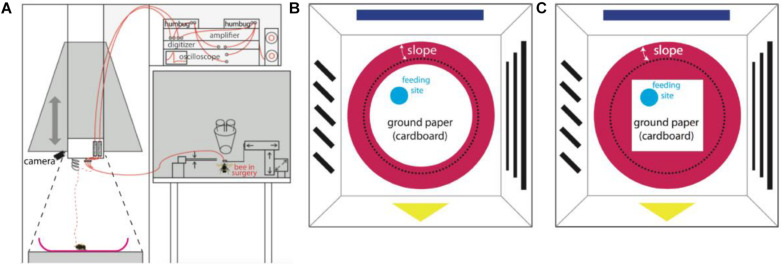
Experimental set-up. **(A)** Recording set-up. A sketch of the whole apparatus modified from Duer et al., 2015. The set-up was composed of an artificial arena environment for behavioral and neural recordings (left half) close to a neural set-up for dissecting the brain, inserting the electrodes, testing for unit activities and for storing neural data (right half). The surgery was done on the table to the right under binoculars. The pyramid hanging over the left part was designed as a Faraday cage and could be lowered down to the ground forming an enclosed space and well shielded. Neural signals were picked up by two twisted 15 μm diameter copper wires and a silver 50 μm diameter ground wire and transmitted to two amplifiers (red lines), an oscilloscope, a pair of loud speakers, an analog-digital converter, and finally stored in a computer. The dashed red line shows the electrode after releasing the bee on ceramic plate, while the black dashed line marks the position when the hanging pyramid was lowered to the ground. **(B)** The bird’s eye view of the arena environment (AE) with round ground paper (when the pyramid containing panorama patterns was on the ground). A video camera mounted on the ceiling recorded the movements of the bee. Simple patterns were pasted on the inner white walls as panorama. A red ceramic plate was placed in the center of the ground. A piece of round white cardboard paper (diameter 22 cm) was fixed to center of the plate; the edge of the white paper was 1 cm away from the lower end of the slope (black dashed line). Fresh sucrose solution (50% in volume) was supplied on a tiny blue feeding disk. The location of the feeding disk was changed from bee to bee, but was always between the center and the edge of ground paper. **(C)** The bird’s eye view of the AE with squared ground paper (diagonal 22 cm). This experimental condition only applied to bee # 150512.

Bumblebee (*Bombus terrestris*) colonies (Schneckenprofi, Hennstedt, Germany) were kept in a glasshouse under controlled temperature ranging from 21 to 27°C. Fresh sucrose solution (30% in volume) and pollen powder were provided daily *ad libitum*. Forager bumblebees were caught at the feeder, and then their right wings were cut so that they could only walk on the bottom of the ceramic plate (Figures 1A–C).

### Behavioral Apparatus

The apparatus (arena environment, AE) was modified for our needs based on a previous description (Duer et al., 2015; Figure 1) with the aim of exposing the searching bumble bee to two different areas, the open field (termed “ground,” including a local cue at a feeding place) and the surrounding slippery slope area that signaled to the animal the border of the arena. In brief, a crimson ceramic plate (rim diameter 35 cm, bottom diameter 24 cm, height of rim 4.5 cm) was placed in the center of a platform (24.5 cm from above the room ground). A round piece of white cardboard paper (diameter 22 cm) was fixed to the center of the ceramic plate surrounded by a crimson-white color contrast boundary (“boundary”). A round piece of blue plastic cardboard (3.5 cm diameter) carrying sucrose solution was placed on the white paper at any location between the center and the edge of ground paper. This blue disk functioned as a local cue to be learned by the bee as a feeding place. The surrounding surface of the plate slope (“slope”) was sprayed with Teflon (dry PTFE Spray, Ballistol, Aham, Germany) such that walking bees could not climb over the rim of the plate.

A wooden dome with a grounded metal mesh hung over the arena and could be lowered down to cover the ceramic plate (Figures 1B,C). In this position it acted as a Faraday cage together with the metal mesh below the ceramic ground plate. The dome was composed of 4 duplications of equal sized trapezoids (short edge 42 cm, long edge 60 cm, height 79 cm). Simple patterns in different colors were pasted on inner walls, serving as external cues (panorama). White and infrared LED matrices were symmetrically arranged on the ceiling of the dome. A web camera (Pro 9000, Logitech, Apples, Switzerland) was fixed on top of the ceiling to record movement of the bumblebee through a small hole in the ceiling. Videos were taken with a frame rate of 10fps and a resolution of 1600 by 1200 pixels. After each recording (ended when units stopped firing), non-paper materials were cleaned with 70% ethanol, the ground paper was exchanged, and Teflon was re-sprayed and dried at room temperature.

### Electrodes and Surgery

The recording diode was constructed according to previous reports (Mizunami et al., 1998a; Okada et al., 1999, 2007). In brief, two 15 μm diameter copper wires (P155, Elektrisola, Hamburg, Germany) were twisted and glued to each other as single-ended electrodes. A 50 μm diameter silver wire (AG548323, Advent Research Materials, Oxford, United Kingdom) was used as reference electrode. The electric resistance of the copper wires was controlled to lower than 50 kΩ by gold plating (Ferguson et al., 2009) as monitored by a digital impedance meter (nanoZ^TM^, White Matter, WA, United States). The diodes were connected to extracellular amplifiers (npi electronic GmbH, Tamm, Germany).

**FIGURE 2:**
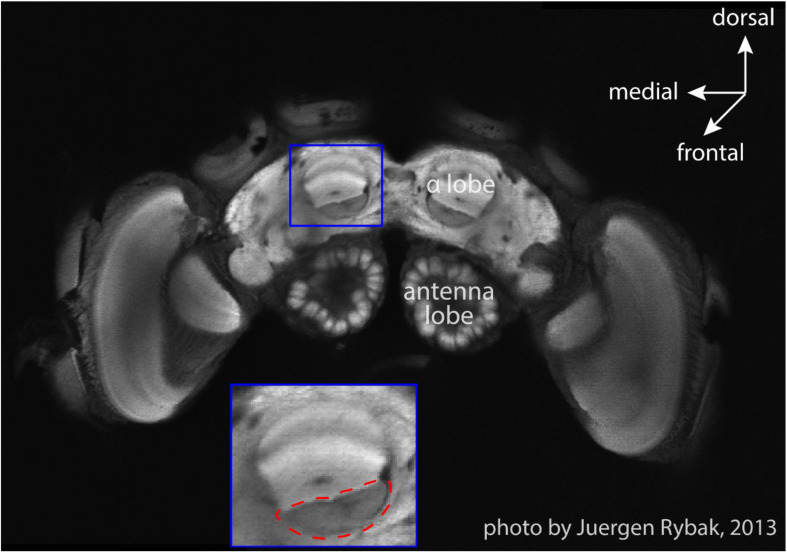
An optical slice of bumblebee brain. The subfigure shows the zoom-in of MB vertical lobe region, and the red dashed-line circle marks the region (β-exit of the MB vertical lobe) where copper electrodes were inserted for neural recordings (courtesy Jürgen Rybak).

The surgery and search for MBENs was performed on a closely attached table outside of the pyramid under an anatomical lens. Single bumblebees were fixed in a metal tube after hypothermic anesthesia, the head was immobilized by clamping the mandibles with forceps, and the antennae were carefully immobilized with Plasticine^®^ (Harbutt, United Kingdom) to reduce disturbance to surgery. A small window was opened in the head capsule with a broken razor blade to expose the position of the right vertical lobe of the MB. The trachea sacks were removed to facilitate the insertion of recording electrodes. The silver electrode was inserted into left ocellus to about 100 μm depth. The diode was slowly inserted into the β-exit region of the vertical lobe (Rybak and Menzel, 1993; Figure 2) with the assistance of a microdrive. The insertion depth was carefully adjusted to 150 μm until stable neural activity was found. To stabilize the position of electrodes, a tiny amount of two-components silicon Kwik-Kast^®^ (World Precision Instruments Company, Sarasota, FL, United States) was filled through the window into the space inside the head capsule. Beeswax was additionally applied to fix the electrodes to the outer surface of head capsule. The experimental bumble bee carrying the recording wires was carefully transferred to the arena, released from the metal tube onto the ceramic plate under red light, and then the dome structure was lowered down to cover the ceramic plate. The recordings in white light were started manually after the bee had been left in darkness for 10 min to recover from surgery.

### Analysis of Neural Activity

The neural activity of single units was manually sorted from multi-units data on the basis of peak-to-trough amplitude and wave shape, and were further confirmed with PCA clustering and event interval analysis, using Spike 2 software (version 7, Cambridge Electronic Design Limited, Cambridge, United Kingdom). Units were included in the analysis if their spike widths (from peak to peak) were longer than 0.6 ms and shorter than 1.2 ms. Recordings longer than 2 min from non-paralyzed bees were included into the database. Customized Matlab (version 2016b, MathWorks, Natick, MA, United States) scripts were written for tracking bee locations from video frames, analyzing neural recordings and statistics. The raw neural data were first organized in a time resolution of 100 ms, and then instant animal locations and neural activity were synchronized by time stamps. Next, bee trajectories were smoothed with three filters: first, single “sharp turns” were corrected. Most tracking errors were only one separated point within a sequence of correct tracking. When the trajectory showed a bee turning left or right >135°, this turning start point (1st point) and the point after turning (3rd point) were directly connected with a line segment, and the 2nd point was re-located as the middle point on this line segment. Second, “jump and stay” were corrected. In rare cases, tracking points jumped to a wrong location and lasted 200–400 ms (2–4 points); therefore, we compared every point with the following 5 points and took the point nearest to the 1st point as the real bee location, the points in between the two real points are evenly re-located between them. In a last step, we applied robust smoothing with a well-established Matlab function SMOOTHN^1^ to polish up the trajectory curves. Furthermore, every bee location was assigned to a certain region (slope, feeding place or ground without feeding plate). The feeding place did not attract enough visiting times; therefore, it was excluded from all analyses.

Spatial information content (IC) was calculated according to well-established methods (Markus et al., 1994), which is used to estimate to what extent the spatial distribution of spike frequency predicts the location or direction of an animal. The IC of the location was calculated based on 19 mm × 19 mm squared bins, while the IC of the angle was based on 6°/sector bins. ICs of three types of angles were tested in every unit: 1. walking direction, 2. target orientation from instant bee location, and 3. target orientation relative to walking direction (i.e., angle 1 – angle 2).

### Experimental Design and Statistical Analyses

To look into the correlation between walking speed and neuro-activity, we first pooled spike numbers in the entire arena by every speed segment of 0.5 cm/s from 0 to 8 cm/s and ran a one-way ANOVA to check the overall spiking difference between speed groups. Next, we compared neural activity on slope and ground separately within each speed group with Wilcoxon rank sum tests followed by Bonferroni correction that controlled type I error from multiple comparisons.

To examine the stability of neuro-activity over time, each recording time was arbitrarily cut into 5-min epochs. Spike frequency was compared between slope and ground within same speed group in the same epoch. Some data sets included sample numbers <30 samples that might lead to less reliable statistic results and thus were excluded from our statistics. Data pairs with big sample numbers (≥30) were compared in independent-samples *t*-tests and *p* values were corrected with Bonferroni correction.

## Results

**FIGURE 3:**
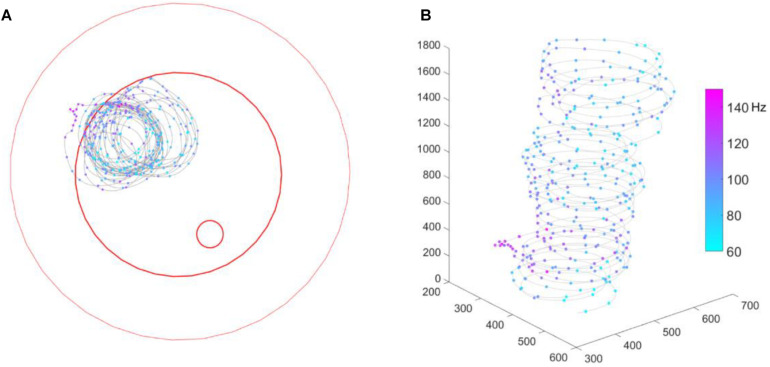
Bee # 140508 running in a circle. **(A)** 2D bee trajectory from bird’s eye view; **(B)** same trajectory in 3D, time goes from down to up. In both sub-figures, the gray line shows the trajectory, each colorful dot on the trajectory shows a down-sampled bee location (per 0.5 s) and spike activity of that moment. The three red open circles from big to small mark the borders of the plate rim, ground paper and feeding disk, respectively.

**FIGURE 4:**
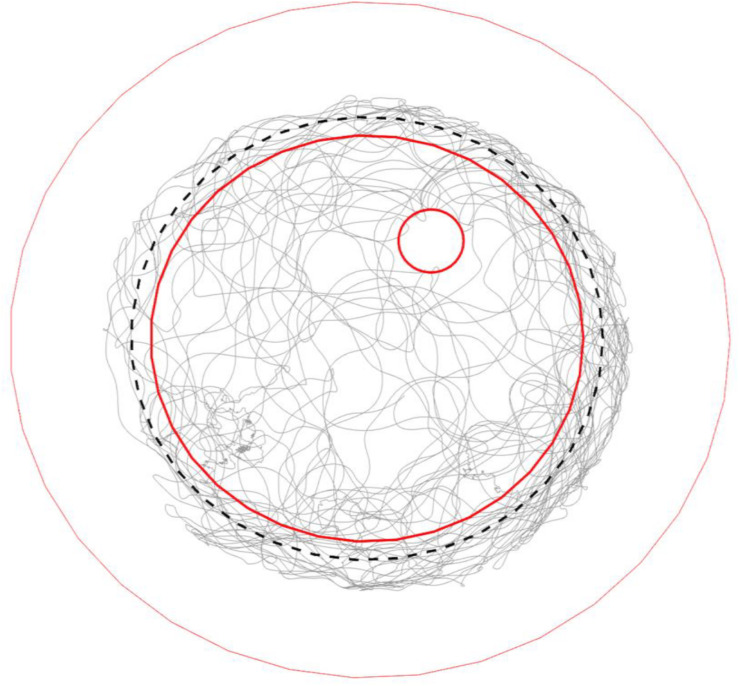
Typical trajectory of a walking bumble bee (#140409) in the arena. Gray lines show the trajectory of a full recording (28 min). The three red open circles from big to small mark the borders of the plate rim, ground paper and feeding disk, respectively. The black dashed-line circle marks the invisible lower end of slope. The bee fully explored the ground area, tried to escape from the slope during thigmotactic walking and visited the feeding place occasionally.

We recorded extracellular neuro-activity of MBENs from 5 bees and sorted out a total of 19 units (see Table 1). Recording durations varied from about 3 min to 2 h, and each bee yielded 1–7 units. During the recording, 4 out of 5 bees walked properly and explored the whole arena. One bee (# 140508) circled counter-clockwise throughout the recording time (2 min 54 s), was only occasionally interrupted by immobility. We include the data of this bee although its behavior was rather unnatural since the corresponding spike activities indicated a behaviorally relevant pattern (Figure 3 and Supplementary Video S1). Interestingly, spike activity increased initially when the animal reached the slope, but this effect disappeared over time. Notice that the spatial components (body direction, location) between ground and slope are correlated with the shift between the two behavioral states, exploration on the ground and escape on the slope. None of the bees visited the feeding place frequently enough to allow a relevant analysis (Figure 4), possibly because they were not hungry enough. We collected the bees from the colony or at the feeder. It is also possible that they were not well adapted to the experimental conditions because they were exposed to the test arena to the first time. This may have led to stronger escape behavior.

**TABLE 1:** All recorded units.

Bee ID	Neuron ID	Record length	Spiking freq. (Hz)		SEM	
			Ground	Slope	Ground	Slope
140409	1	28 min	26.10	30.95	0.17	0.22
	2	28 min	13.89	22.99	0.23	0.30
	3	28 min	9.62	12.30	0.13	0.19
	4	28 min	11.56	13.09	0.11	0.15
	5	28 min	8.02	10.38	0.12	0.18
	6	28 min	1.30	2.10	0.04	0.06
	7	28 min	0.53	0.99	0.03	0.04

140508	1	2 min 54 s	93.24	107.27	0.91	3.88

141204	1	59 min 57 s	15.10	17.71	0.07	0.13
	2	59 min 57 s	14.16	12.18	0.06	0.12
	3	59 min 57 s	13.53	11.30	0.06	0.09
	4	59 min 57 s	11.02	16.15	0.09	0.20
	5	59 min 57 s	5.44	8.10	0.04	0.09
	6	60 min	1.70	1.47	0.02	0.03
	7	60 min	7.91	6.14	0.06	0.08

141217	1	120 min 1 s	4.42	7.13	0.04	0.09
	2	120 min 1 s	14.34	23.51	0.12	0.18
	3	120 min 1 s	12.99	10.80	0.09	0.12

150512	1	59 min 55 s	6.35	4.98	0.07	0.04

*In total 19 units were sorted out from 5 bumblebees; each bee yielded 1–7 units and each unit was recorded for ∼3 min – 2 h. Mean spiking frequency and SEM of whole recordings on ground and slope are listed separately.*

**FIGURE 5:**
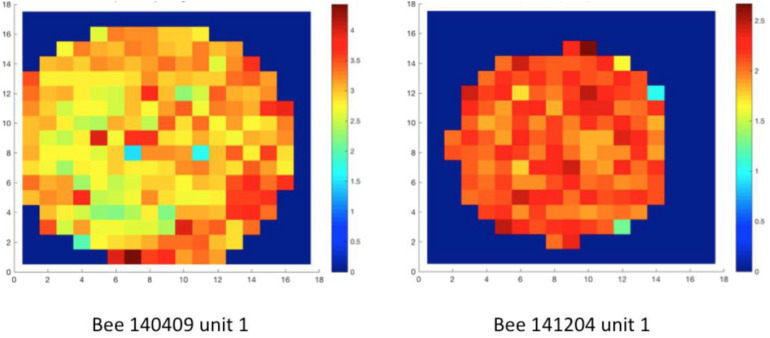
Spatial distribution of neural activity of two units in two different animals. The whole arena was divided into squared bins (each bin 19 mm × 19 mm). False colors represent neuro-activity within each spatial bin, which is the mean spike frequency (Hz) at all tracking points in each bin. Spatial information content (IC) was calculated based on well-established methods (Markus et al., 1994). The ICs of unit 1 in bee # 140409 and unit 1 in bee # 141204 were 0.009 and 0.003, indicating no location-predictive (or so-called “place cell”) property in these units. Data of other units are in Supplementary Figure S1.

**FIGURE 6:**
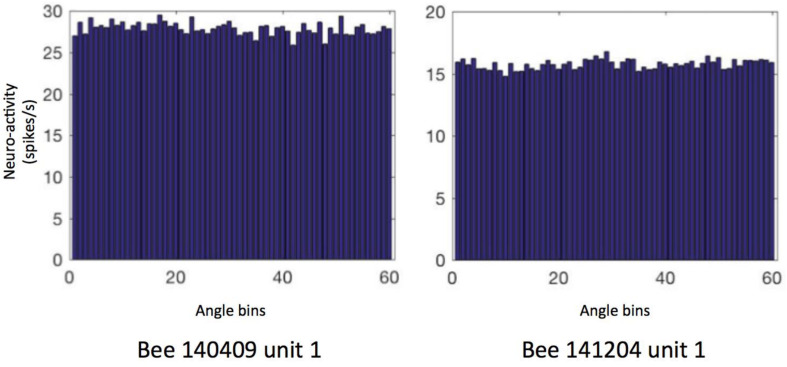
Heading direction related neural activity in two neurons. The spiking frequency in 360° of arena is binned into 60 sectors (i.e., 6°/sector) and then averaged. Data of other units are in Supplementary Figure S2. The directional ICs of unit 1 in bee # 140409 and unit 1 in bee # 141204 are 0.0004 and 0.001, indicating no direction-predictive (or so-called “head direction cell”) property in these neurons.

First, we focused on the neural activity during walking on the ground. We checked if the neuron firing patterns can stably predict location (unit examples in Figure 5) or head direction (unit examples in Figure 6). Therefore, we calculated the so called “information content (IC)” of our recorded units referring to the method in rodents place cell analysis (Markus et al., 1994). It turned out that our IC values varied from 0.009 to 0.3, lower than the criterion of 1 as a sign of place cell, indicating that our recorded units did not contain place cell-like properties (Supplementary Figure S1). Similarly, no head direction cell-like properties were found, with IC values ranging from −0.0002 to 0.1; target orientation IC ranged from −0.27 to 0.6; heading direction away from target (i.e., head direction- target orientation) IC varied from −0.03 to 0.12 (Supplementary Figure S2).

### Correlation Between Walking Speed and Neural Activity

**FIGURE 7:**
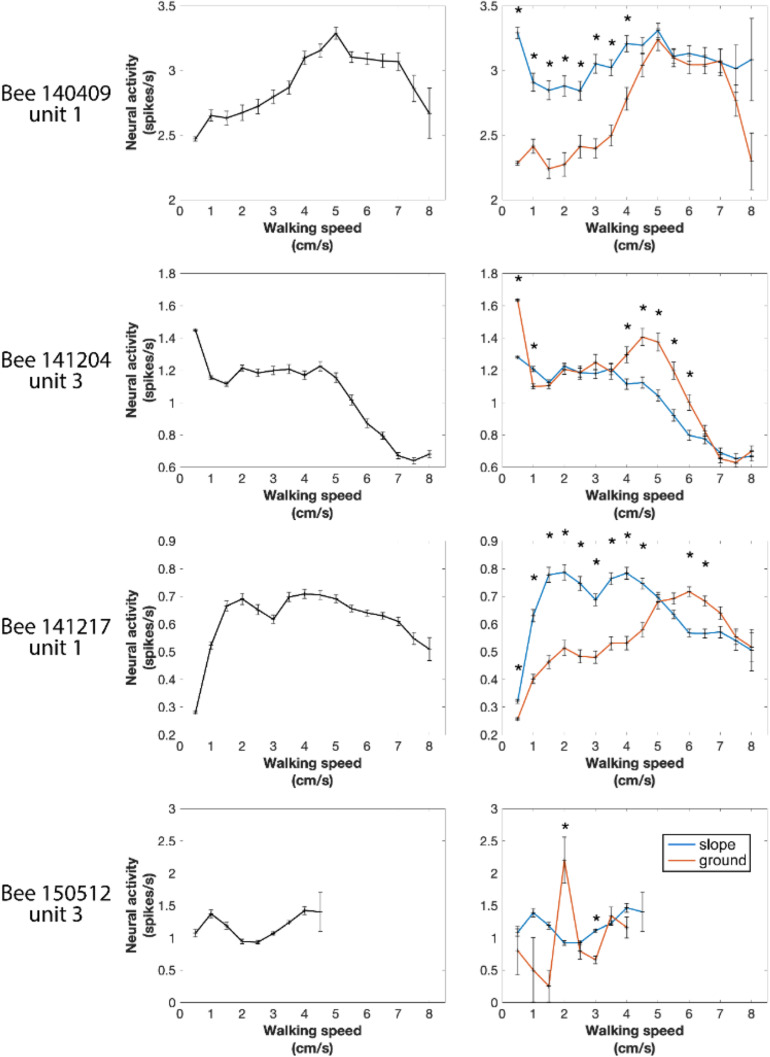
Four examples of neural activity at different walking speeds in the entire arena and in the two different compartments of the arena (ground and slope). Spike numbers were pooled by every speed segment of 0.5 cm/s from 0 to 8 cm/s and in each speed group normalized to 1 s. **Left panels:** entire arena. One-way ANOVA showed statistically significant difference in every recorded neuronal unit (all *p* values < 0.001), indicating that in each neuron at least two speeds were correlated with different neural activity. **Right panels:** neural activity separately calculated for different walking speeds on the slope and the ground. Units were differently active when the bee walked on the two compartments of the arena at some speeds but not all. *p < 0.05 in Wilcoxon rank sum tests with Bonferroni correction.

Next, we tested the overall correlation between walking speed and neural activity. One-way ANOVA showed statistically significant differences in every recorded unit (all *p* values < 0.001), indicating that neural activity between at least two speed groups in each unit was significantly different. However, for individual units, the highest neuronal activity could appear at any speed (see examples in Figure 7 left panels and Supplementary Figure S3). These results do not indicate that the recorded units were directly controlling walking speed because the sensory and central processing components (both internal and external conditions) leading finally to changed walking speed may be the parameters influencing the units’ activities. Therefore, we looked into parameters related to the parameters of the arena that the bees experienced during changes in spike activity.

In our first approach we asked whether the units were differently active on ground and slope, since the two areas had different textures (paper vs. ceramics), colors (while vs. magenta) and features related to gravity, and they were obviously differently related to two behavioral states, exploration and thigmotactic escape. To this end, we further divided the spike data between ground and slope, and compared them with Wilcoxon rank sum tests. Because the walking speed distributions on the two structures were significantly different in every recording of natural walk (Supplementary Figure S4), we first normalized the neuro-activity in each narrow speed bin (step = 0.5 cm/s) to the same time unit (spikes/s). As in the initial phase of the circling bee # 141508 (Figure 3), all units were activated differently when they were on ground and slope. This effect depended on walking speed in different ways for different animals (Figure 7 right panels and Supplementary Figure S3). These results clearly show local effects on neural activity possibly related either to area within the whole arena or the two behavioral states related to these two areas (exploration and thigmotaxis).

**FIGURE 8:**
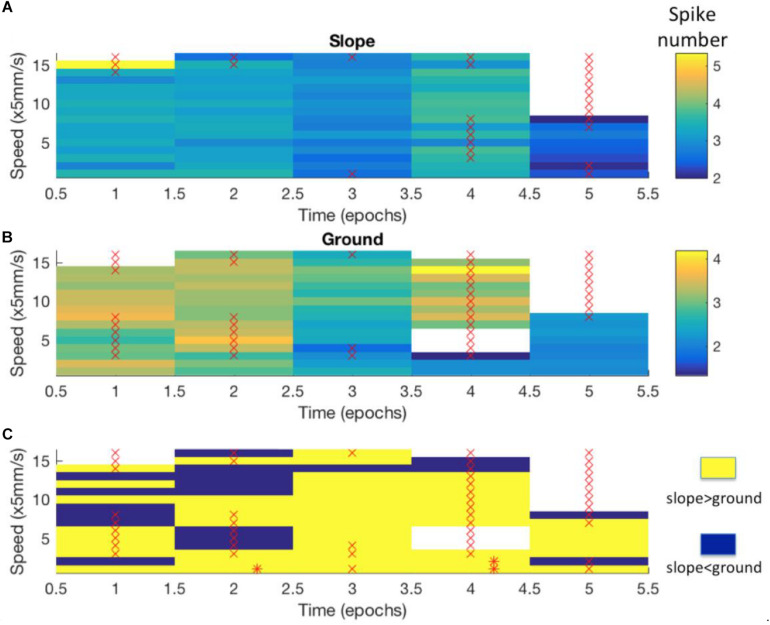
Differences in neuro-activity between slope and ground at a certain speed were not constant over time. The example shown here stems from Bee 140409, unit 1. Other examples are shown in Supplementary Figure S5. The whole recording time was cut into 5-min epochs. **(A)** Neuro-activity along time (abscissa) when the bee was on the slope walked at different speed (ordinate). Spike activity is expressed in false color as indicated in the right upper corner. **(B)** The same graph for the bee walking on the ground. **(C)** Comparison of time dependence of spike activity between slope and ground. Higher spike activity on the slope is expressed in yellow, and higher spike activity on the ground in blue. * marks significant differences between slope and ground (*p* < 0.05 in independent-samples *t*-tests after Bonferroni correction). × marks low sample numbers (<30) that lead to less reliable statistic results and are thus excluded from our statistics.

Mushroom body extrinsic neurons are known to respond to multiple sensory conditions in a combinatorial way and they are sensitive to the context in which stimuli appear (Filla and Menzel, 2015; Zwaka et al., 2018). This latter effect could possibly reflect state-dependent activities. Therefore, we went on to test whether the effects we found for the region-speed dependence reflected a consistent property or varied over time. The latter was found. The whole recording time of each unit was arbitrarily binned to 5-min epochs. All recorded units (Table 1) that showed different spike activities between slope and ground at the same walking speed changed these properties over time, either within each region (Figures 8A,B) or with differences of neural activity between slope and ground (Figure 8C). For other units, see Supplementary Figure S5. Taken together with the results in Figure 7, the recorded MBENs appeared to combine several conditions the actively exploring animal finds itself in. The two areas, ground and slope, differed with respect to their spatial arrangement and their meaning to the animal, open area for exploration and boundary of a restricted area that the animal may want to leave (thigmotactic escape). The two areas were well characterized by different walking trajectories, flexibly changing walking directions on the ground and stereotypical trajectories when on the slope (Figure 4). The next step will be to analyze repetitive behavioral elements that are consistently related to neural activity changes. Are these epochs similarly or differently related to spike activity changes in the ground and slope area?

**FIGURE 9:**
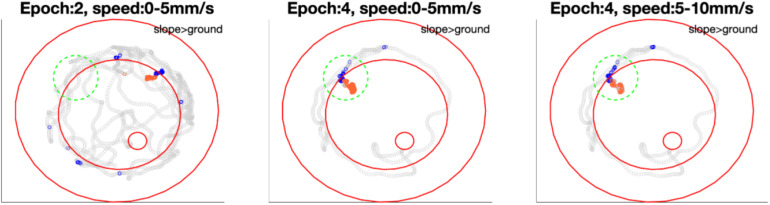
Bee 140409 locations in * marked epochs of Figure 8. In each subfigure, blue points highlight the bee locations on the slope in that epoch under certain speed, while orange points highlight locations on the ground in the same epoch and same speed. Gray points show other locations of the same epoch in all speeds. Three red open circles, from small to big, mark the feeding place, edge of ground paper and rim of arena. The comparison of mean spike numbers between blue and orange points is displayed in the up-right corner of each subfigure. Note that in the area marked with a green dashed circle, the unit was not significantly more active in epoch 2 on either slope or ground, however, it was more active on the slope when the bee revisited this area in epoch 4. Different significant locations in different epochs indicate that in Figure 8 the inconstant difference over time is caused by time instead of revisiting of the same location. More examples of other units are in Supplementary Figure S6.

**FIGURE 10:**
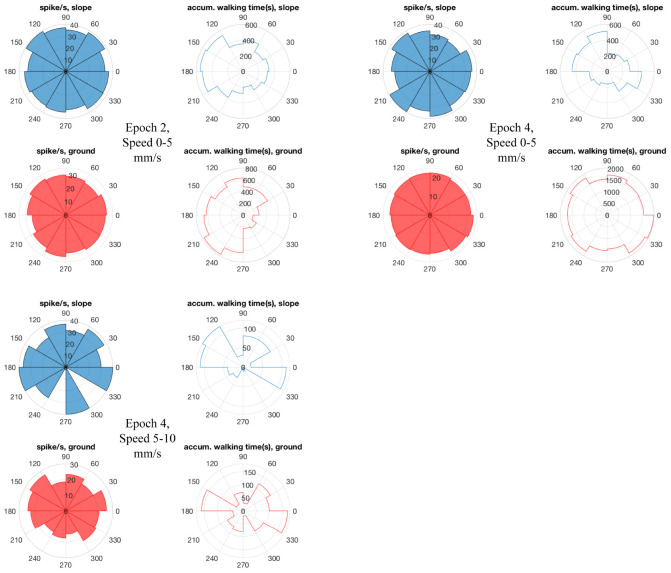
Bee 140409 walking directions and neuro-activity in * marked epochs of Figure 8. The 360° is binned into 12 sectors (i.e., 30°/sector). Blue sectors: slope, red: ground. Filled sectors: spikes/second; unfilled: accumulated walking time. Data of other units are in Supplementary Figure S7.

**FIGURE 11:**
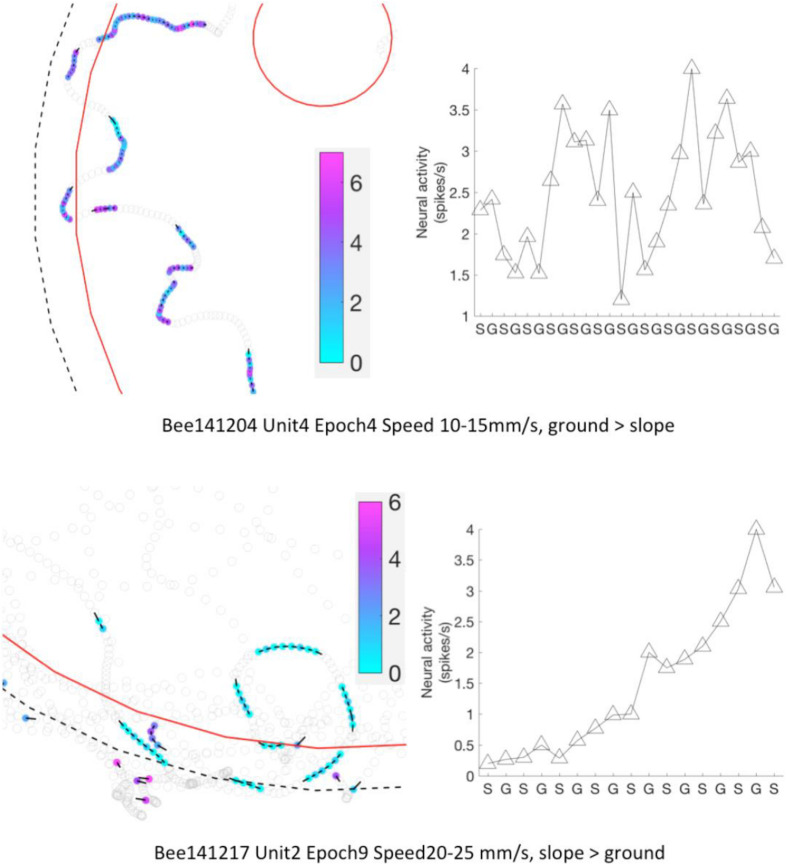
Neural activity of MBENs accumulated for each significant epoch of walking between slope and ground. Each row shows one significant epochs from two bees. **Left panels:** examples for parts of the arena that included frequent region shifts of the bee between slope and ground. Bee locations in a significant epoch are highlighted with color dots; neural activity (spikes/s) from low to high is represented from light blue to magenta (color bar). Other bee locations are marked with open circles. Black bars point to heading direction at that tracking point. **Right panels:** mean spike numbers on slope and ground during region shifts in the same sig. epoch as **left panels**. S: slope; G: ground.

Next we examined the epochs where neural activity was significantly different between slope and ground explorations at a particular walking speed (mentioned as “sig. epochs” below, marked with ^∗^ in Figure 8C). Three hypotheses were tested: (1) Was the bee at the same location in different sig. epochs? (2) Was the bee facing the same direction in different sig. epochs? (3) Within each sig. epoch, was the neuro-activity on slope constantly higher or lower than on ground? We found that the same unit in different sig. epochs could be activated at different locations (one example in Figure 9) and had no preferred heading direction in any sig. epoch (one example in Figure 10), meaning that the inconsecutive sig. epochs resulted from varying neural activity over time rather than returning to a certain spatial location or heading direction. Furthermore, within any sig. epoch the neural activity was not always higher on the slope than on the ground (or the other way round) over time (examples see Figure 11), altogether indicating that the different neural activity of MBENs between slope and ground in sig. epochs was a combinatorial and accumulative effect, rather than a single-property or stably spiking in small time scales under the same conditions.

To conclude, the neural activity of all recorded MBENs did not correlate with spatial parameters (location, direction), neither on a macro time scale (up to ∼2 h of whole recording) nor on a micro scale (a few seconds in sig. epochs); instead, they reflected in a walking tempo-dynamic way different behavioral states when the bees were actively exploring on ground or thigmotactically walking on slope.

## Discussion

The search for neural correlates of active exploration in small animals like the bumble bee imposes inevitable compromises with respect to the kind of movement (walking vs. flying), the size of the test arena, the motivation of the animal and the confinement imposed on the animal by the border of the arena. Our emphasis was to replicate as much as possible behavioral studies that documented operant learning of a local color cue at a feeding site and the visual pattern of the panorama characterizing the feeding site (Jin et al., 2014). The animals in these experiments were fasted overnight before every training and test, therefore, explored actively the environment and searched for the location of a feeding place both in relation to its local cue and the panorama. The bumble bees relied on spatial relations of the visual features within the test arena as shown by the effects caused by rotating the panorama and displacing the local cue. Other than in these former studies the bees in the experiments reported here were brought to the arena directly from the colony or a feeding place without overnight fasting, and thus they might have been less motivated to feed. They did not suck sucrose solution at the feeder and did not return multiple times to it although they explored the whole arena extensively. We were not concerned about the lack of motivation to feed in the arena because our aim was to search for neural correlates at the level of the MBENs during exploratory behavior as an essential requirement for navigational performance. Animals learn during exploration (Tolman, 1948), a form of learning that has been documented for honeybees in the context of navigation under natural conditions (Degen et al., 2015, 2016).

We aimed to record from MBENs because these neurons read out the high order neural processing in the MB, a structure of the insect brain well known for its convergence of highly processed sensory information and its role in memory formation (Heisenberg, 2003; Menzel, 2012). The MB consists of a large number (∼150,000) of densely packed small neurons (Kenyon cells, KC) that converge on a small number (a few hundred) ENs (Rybak and Menzel, 1993). Other than KC, ENs respond to stimuli of multiple sensory modalities indicating a different coding scheme than the highly specific combinatorial sensory code at the input of the MB (Menzel, 2012). All MBENs so far recorded change their response properties during associative learning. For example, a large EN, the peduncle extrinsic neuron #1 (PE1), was found to reduce its responses to the learned odor (Mauelshagen and Greggers, 1993; Okada et al., 2007) but enhances its responses to a visual context stimulus that signaled to the animal the upcoming learned stimulus. These results indicated that PE1 encodes cues and contexts differently, possibly representing a general property of ENs.

The MB is characterized by prominent recurrent neurons (Zwaka et al., 2019) whose neural activity relates to novelty detection, context dependence, and expectation (Filla and Menzel, 2015). ENs increase their responses to the learned stimulus, decrease them or do not change. In most cases the range of multi-sensory responses characteristic for the respective neuron is broad and rather unspecific before learning and becomes more specific after learning (Strube-Bloss et al., 2011, 2016). For example, half of a subset of ENs (the A1, A2 neurons) change their responses to the reinforced stimuli, the other half continued with stable responses. Most of the plastic neurons change their responses not during the acquisition process but after a consolidation phase of a few hours. Two kinds of changes were observed, qualitative changes (switching) and quantitative changes (modulating). Switching neurons dropped responses and/or developed new responses to one or several of the tested odors. All switches observed with respect to the CS+ odor were recruitments; those to the CS− could have been either recruitment or loss of response. Modulating neurons, however, increased and/or decreased their response rates to different odors.

Most likely, response properties of high order interneurons depend on whether the animals are passively exposed to the stimuli or actively exploring them. Indeed, MBENs were found to change their responses to visual and olfactory stimuli under operant learning conditions in a virtual reality environment (Zwaka et al., 2018). Furthermore, MBENs were found to predict responses in active social contexts that allowed the recorded animal to move freely within the community of a small colony (Duer et al., 2015; Paffhausen et al., 2020). Thus the MB as a whole appears to act as a re-coding device, converting sensory information to value-based information that provides the information for active behavioral control (Menzel, 2014). In this respect the mushroom body shares properties with the mammalian hippocampus (Johnson et al., 2007; Yanike et al., 2009; van der Meer et al., 2010) and prefrontal cortex (Histed et al., 2009; Mansouri et al., 2009). As in any other neural system we do not yet understand the logic of the multiple faceted coding and storing schemes composing higher order neural integration but the limited number and the unique structures of ENs in the bee brain offers an opportunity to gain insight.

One reason for our ignorance about high order integration processes is the lack of information about the internal states of the brain. Animal behavior is highly influenced by internal states and thus may lead to changing correlations between neural activity patterns of particular neurons and behavioral acts. Even well-characterized and identified neurons in rather small nervous systems like that of snails, annelids and arthropods change their spiking activities under state-dependent conditions of the brain or lead to different behaviors when selectively stimulated in different contexts. For example, a command-like neuron in the leech that releases swimming in water when intracellularly stimulated induces crawling when the leech is placed on solid ground (Esch et al., 2002). The feeding network in *Aplysia* undergoes network-specific changes depending on its history of activity in different contexts indicating a form of task-specific inertia (Proekt et al., 2004). Stimulation of a flight-inducing command neuron in insects will lead to flight only in air-suspended animals but not in animals placed on the ground, e.g., in crickets (Nolen and Hoy, 1984). In some cases state-dependence of the brain can be traced to neuro-modulatory circuits controlled by the environment, e.g., in insects (Pflüger, 1999; Cohn et al., 2015) but the activity of modulatory systems during recordings of processing neurons is mostly unknown. Hippocampal place cells are not only selectively activated by the local cues of the experienced environment (Moser et al., 2008) but also by the value and meaning of localized olfactory stimuli (Manns and Eichenbaum, 2009), but again it is not known how the selective read-out of the multiple features are controlled. Correlating the activity of single cortical neurons in perceptual or motor tasks is notoriously difficult because of the multiple recurrent networks. What appears as noise may even be the signs of information processing (Engel and Singer, 2001; Salinas et al., 2001; Davis et al., 2019). From a more global perspective, the brain may not require defined external conditions for selecting behavioral acts but rather generates the conditions internally (Gallistel, 2013).

Given these limitations of an electrophysiological approach to high-order brain functions we may not be surprised that correlations between the current stimulus conditions and ongoing or future behavioral acts may fail to be uncovered. Here we applied a rigid selection scheme in our search for correlations. The criterion was that the correlation should stay stable over the whole recording time. This is indeed a sharp tool. No selective place-dependent or body direction-dependent spiking changes were found. Walking speed dependence differed in the two main areas of the arena, ground and slope. Walking along the slope is likely related to thigmotaxis/escape, whereas walking on the ground reflects exploratory behavior. Thus remaining in one of the two areas may relate more to behavioral states than to locations. In such a case the units‘ differences in their walking speed dependence indicates behavioral states rather than location dependence, an interpretation supported by the finding that combinations with other spatial parameters (restricted location, body direction) did not lead to any significant effects.

## Data Availability Statement

All datasets presented in this study are included in the article/Supplementary Material.

## Author Contributions

RM designed the experiment. BP built the setups and did spike-sorting. NJ did bee surgeries, ran experiments, analyzed data, and prepared all table and figures. AD and NJ wrote codes for video tracking of bee locations. RM and NJ wrote the manuscript. All authors contributed to the article and approved the submitted version.

## Conflict of Interest

The authors declare that the research was conducted in the absence of any commercial or financial relationships that could be construed as a potential conflict of interest.

## Footnotes

1 https://www.mathworks.com/matlabcentral/fileexchange/725-smoothn
