# Supplementary figures and images for: Mushroom Body Extrinsic Neurons in Walking Bumblebees Correlate With Behavioral States but Not With Spatial Parameters During Exploratory Behavior
Source: Front Behav Neurosci. 2020 Oct 20;14:590999. doi: 10.3389/fnbeh.2020.590999 (PMC7606933; doi:10.3389/fnbeh.2020.590999)

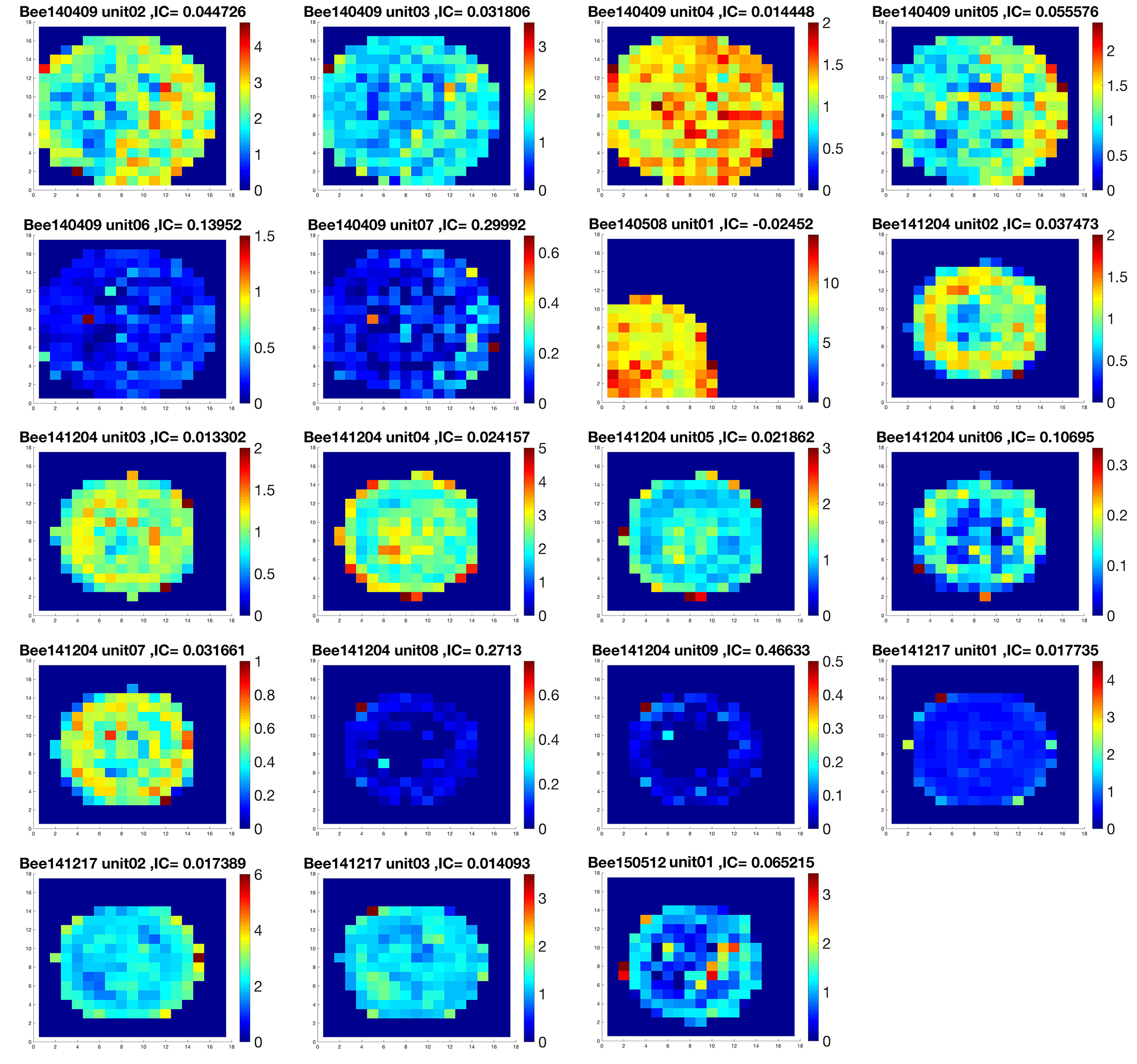

Supplement: Supplementary file 1 [file Image_1.TIF]

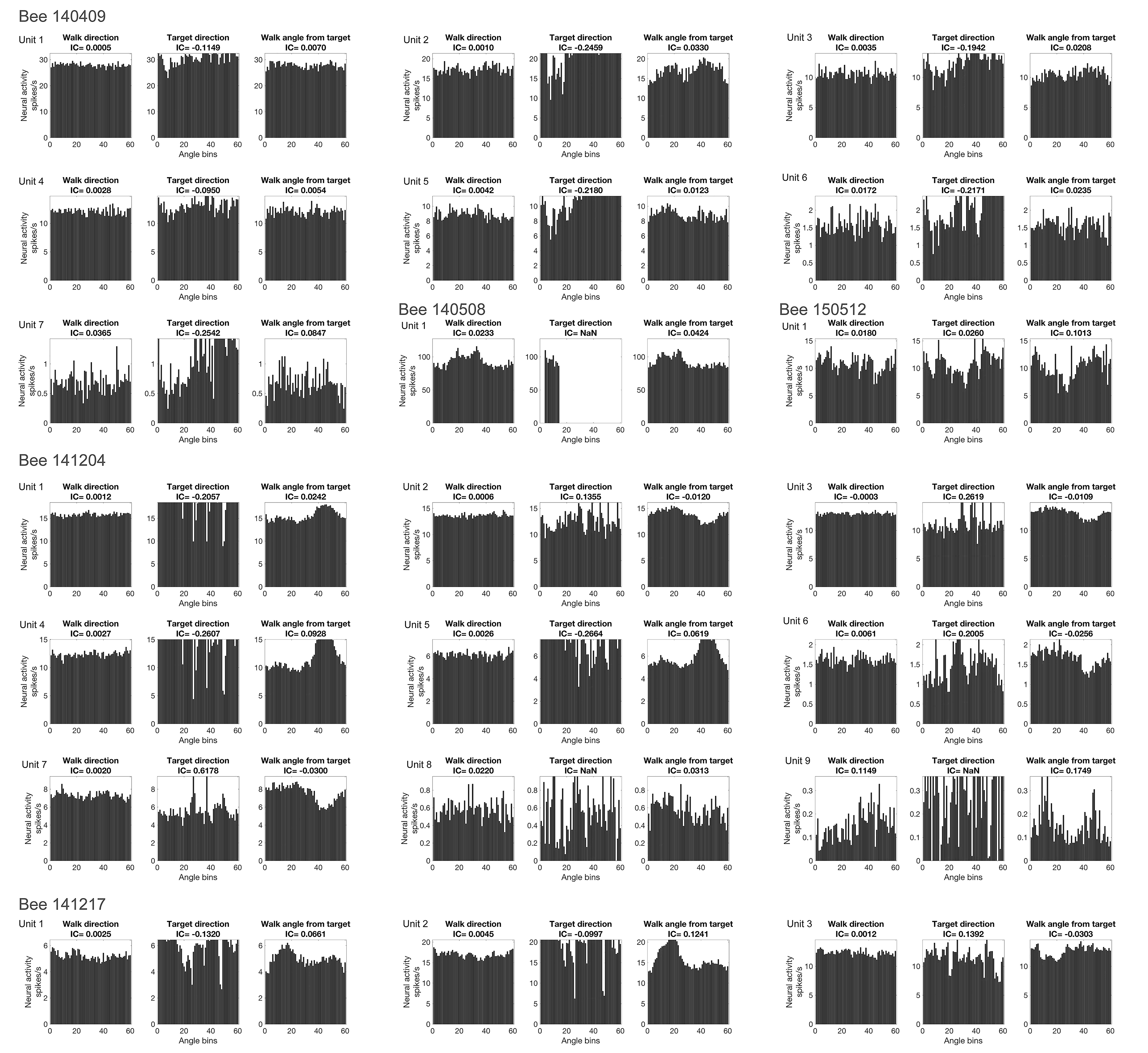

Supplement: Supplementary file 2 [file Image_2.TIF]

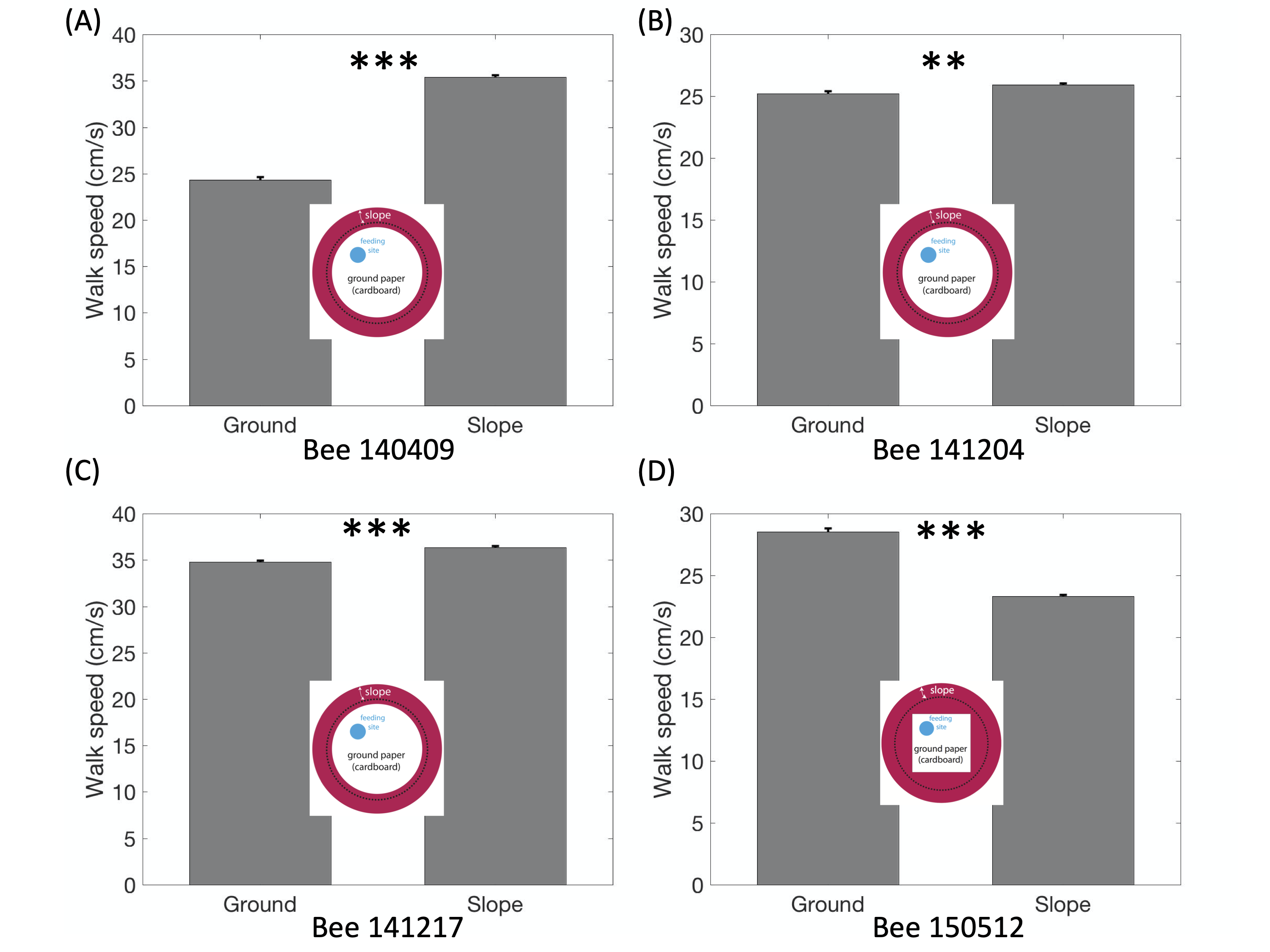

Supplement: Supplementary file 4 [file Image_4.TIFF]

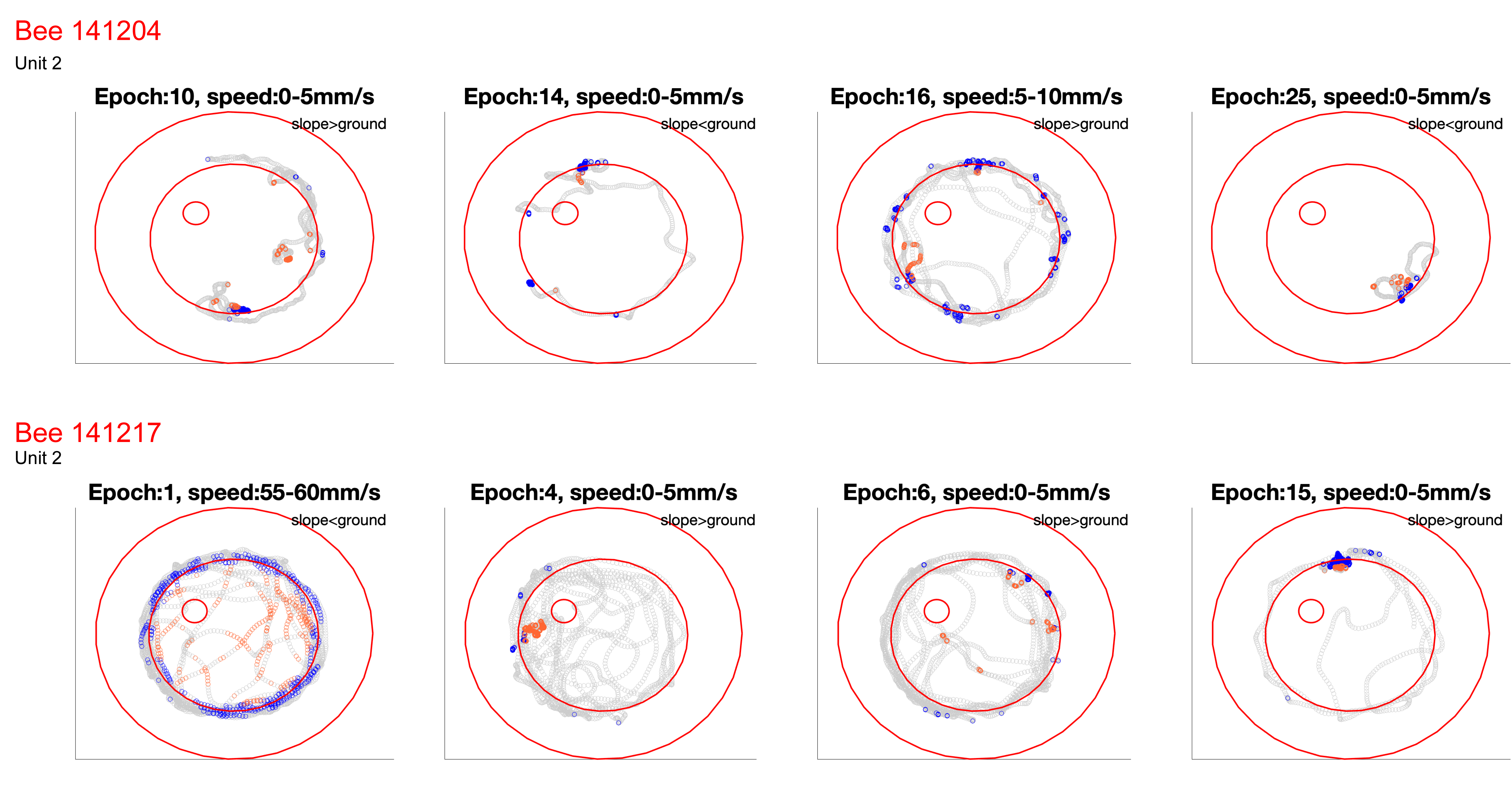

Supplement: Supplementary file 6 [file Image_6.TIF]

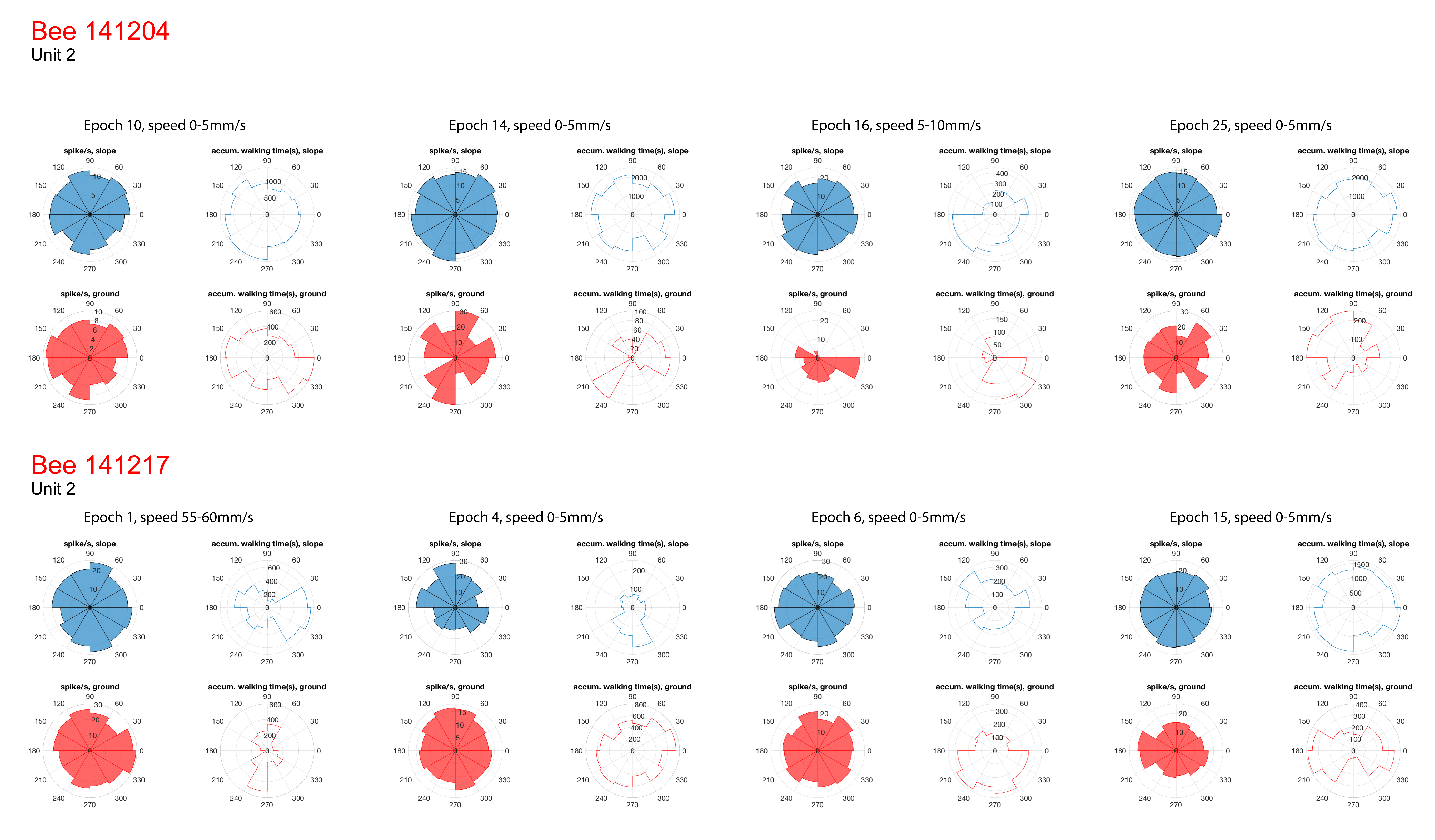

Supplement: Supplementary file 7 [file Image_7.TIF]
