# Supplementary material for: Mushroom Body Extrinsic Neurons in Walking Bumblebees Correlate With Behavioral States but Not With Spatial Parameters During Exploratory Behavior
Source: Front Behav Neurosci. 2020 Oct 20;14:590999. doi: 10.3389/fnbeh.2020.590999 (PMC7606933; doi:10.3389/fnbeh.2020.590999)
Supplement: Supplementary file 8 [file Table_2.DOCX]

**Supplementary Figure 1.** Spatial distribution of neural activity of different units. The whole arena was divided into squared bins (each bin 19x19mm). False colors represent neuro-activity within each spatial bin, which is the mean spike frequency (Hz) at all tracking points in each bin. Spatial information content (IC) was calculated based on well-established methods (Markus et al., 1994). No location-predictive (or so-called "place cell") property was found in these units.

**Supplementary Figure 2.** Heading direction related neural activity in different units. The spiking frequency in 360*°* of arena is binned into 60 sectors (i.e. 6*°*/sector) and then averaged. No direction-predictive (or so-called "head direction cell") property was found in these neurons.

**Supplementary Figure 3.** Neural activity at different walking speeds in the entire arena and in the two different compartments of the arena (ground and slope). Spike numbers were pooled by every speed segment of 0.5 cm/s from 0-8cm/s and in each speed group normalized to 1s. Left panels: entire arena. One-way ANOVA showed statistically significant difference in every recorded neuronal unit (all p values <0.001), indicating that in each neuron at least two speeds were correlated with different neural activity. Right panels: neural activity separately calculated for different walking speeds on the slope and the ground. Units were differently active when the bee walked on the two compartments of the arena at some speeds but not all. *p<0.05 in Wilcoxon rank sum tests with Bonferroni correction.

**Supplementary Figure 4.** Mean walking speed on ground (round or squared) and slope. Three bees walked faster in the slope area than round ground (A, B, C), while one bee faster on the squared ground than slope (D). Speed data from 0-1cm/s were excluded from this test to avoid dilution effect of long rest to the average speed. **p<0.01, ***p<0.001 in independent samples t-test.

**Supplementary Figure 5.** Differences in neuro-activity between slope and ground at a certain speed were not constant over time. The whole recording time was cut into 5-minute epochs. A: Neuro-activity along time (abscissa) when the bee was on the slope walked at different speed (ordinate). Spike activity is expressed in false color as indicated in the right upper corner. B: The same graph for the bee walking on the ground. C: Comparison of time dependence of spike activity between slope and ground. Higher spike activity on the slope is expressed in yellow, and higher spike activity on the ground in blue. * marks significant differences between slope and ground (p<0.05 in independent-samples t-tests after Bonferroni correction). × marks low sample numbers (< 30) that lead to less reliable statistic results and are thus excluded from our statistics.

**Supplementary Figure 6.** Bee 141204 and bee 141217 locations (red: slope, green: ground) in * marked epochs of Fig. 8. Different significant locations in different epochs indicate that the inconstant difference is caused by time instead of revisiting of the same location.

**Supplementary Figure 7.** Bee 141204 and bee 141217 walking directions and neuro-activity in * marked epochs of Fig. 8. The 360*°* is binned into 12 sectors (i.e. 30*°*/sector). Blue sectors: slope, red: ground. Filled sectors: spikes/second; unfilled: accumulated walking time.

**Supplementary Video 1.** Bee # 140508 running in a circle (2D trajectory from bird's eye view). The black line shows the trajectory, each colorful dot on the trajectory shows a down-sampled bee location (per 0.5s) and spike activity of that moment. The three red open circles from big to small mark the borders of the plate rim, ground paper and feeding disc, respectively.
